# Granular cell tumor presenting as a tongue nodule: two case reports

**DOI:** 10.1186/1752-1947-6-56

**Published:** 2012-02-10

**Authors:** Nivea Cristina Sena Costa, Fernanda Bertini, Yasmin Rodarte Carvalho, Janete Dias Almeida, Ana Sueli Rodrigues Cavalcante

**Affiliations:** 1 Department of Bioscience and Oral Diagnosis, São José dos Campos Dental School, São José dos Campos, UNESP - Univ Estadual Paulista, São Paulo, Brazil

## Abstract

**Introduction:**

Granular cell tumor is an uncommon neoplasm that can occur in any part of the body, including the orofacial region. The tumor is usually benign, but there are reports of cases in which the tumor shows a locally aggressive behavior, malignancy, and distant metastases. The most widely accepted hypothesis is that granular cell tumor arises from the altered metabolism of Schwann cells. The tumor is typically asymptomatic and appears as a nodule that does not exceed 3 cm.

**Case presentation:**

In case 1, a 26-year-old Caucasian man was seen at the Oral Medicine out-patient clinic of the São José dos Campos Dental School, Universidade Estadual Paulista, with a 'small blister on the tongue', which he had noted approximately three years ago. The nodule was located on the dorsum of the tongue, measured about 1.5 cm in diameter, and was not tender to palpation. Treatment consisted of an excisional biopsy performed on the basis of the diagnostic hypothesis of granular cell tumor, which was confirmed by microscopic analysis. In case 2, a 31-year-old Caucasian woman attended the out-patient clinic of the São José dos Campos Dental School, Universidade Estadual Paulista, with a five-year history of a 'painful lump on the tongue'. Intra-oral examination revealed the presence of a nodular lesion measuring approximately 0.8 cm in diameter, which was located deep in the submucosa of the right lateral margin of the tongue. Treatment consisted of an excisional biopsy performed on the basis of the differential diagnosis of neurofibroma and granular cell tumor. Microscopic analysis defined the final diagnosis of granular cell tumor.

**Conclusions:**

Granular cell tumor is an uncommon tumor that must be carefully diagnosed and treated correctly.

## Introduction

Granular cell tumor (GCT) is an uncommon benign neoplasm, first described by Abrikossoff in 1926 [1]. The tumor was initially called 'granular cell myoblastoma' due to its possible proposed origin from skeletal muscle. Various theories on the origin of GCT have subsequently been proposed, including its origin from striated muscle and histiocytes and a neural origin.

Granular cell tumors can affect any organ or region of the body. Most GCTs occur in the head and neck region, especially in the tongue, cheek mucosa, and palate [2]. We report here two cases of GCT located on the tongue.

## Case presentation

### Case 1

**Figure 1:**
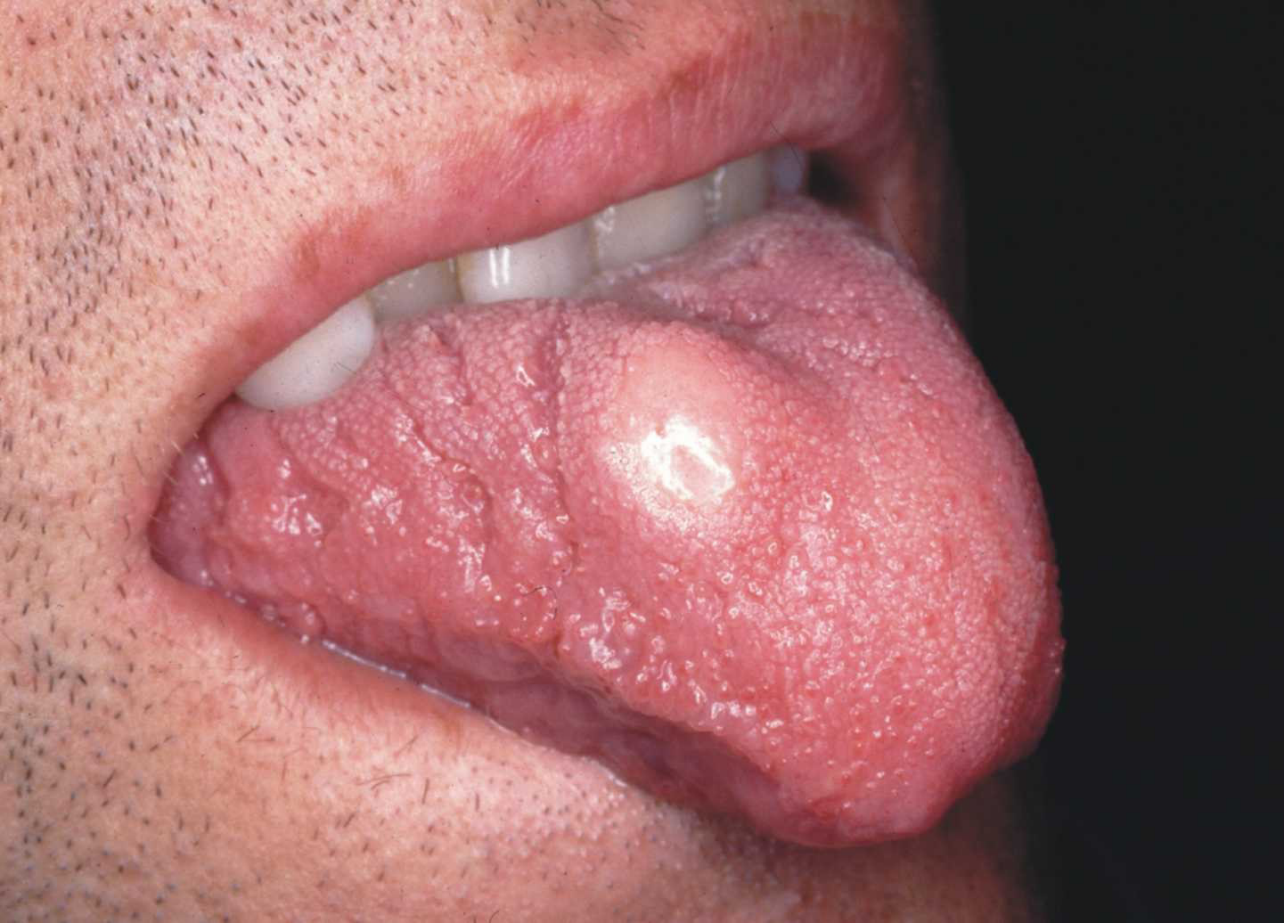
**Well delimited nodular lesion located on the dorsum of the tongue and measuring about 1.5 cm across its major diameter**.

**Figure 2:**
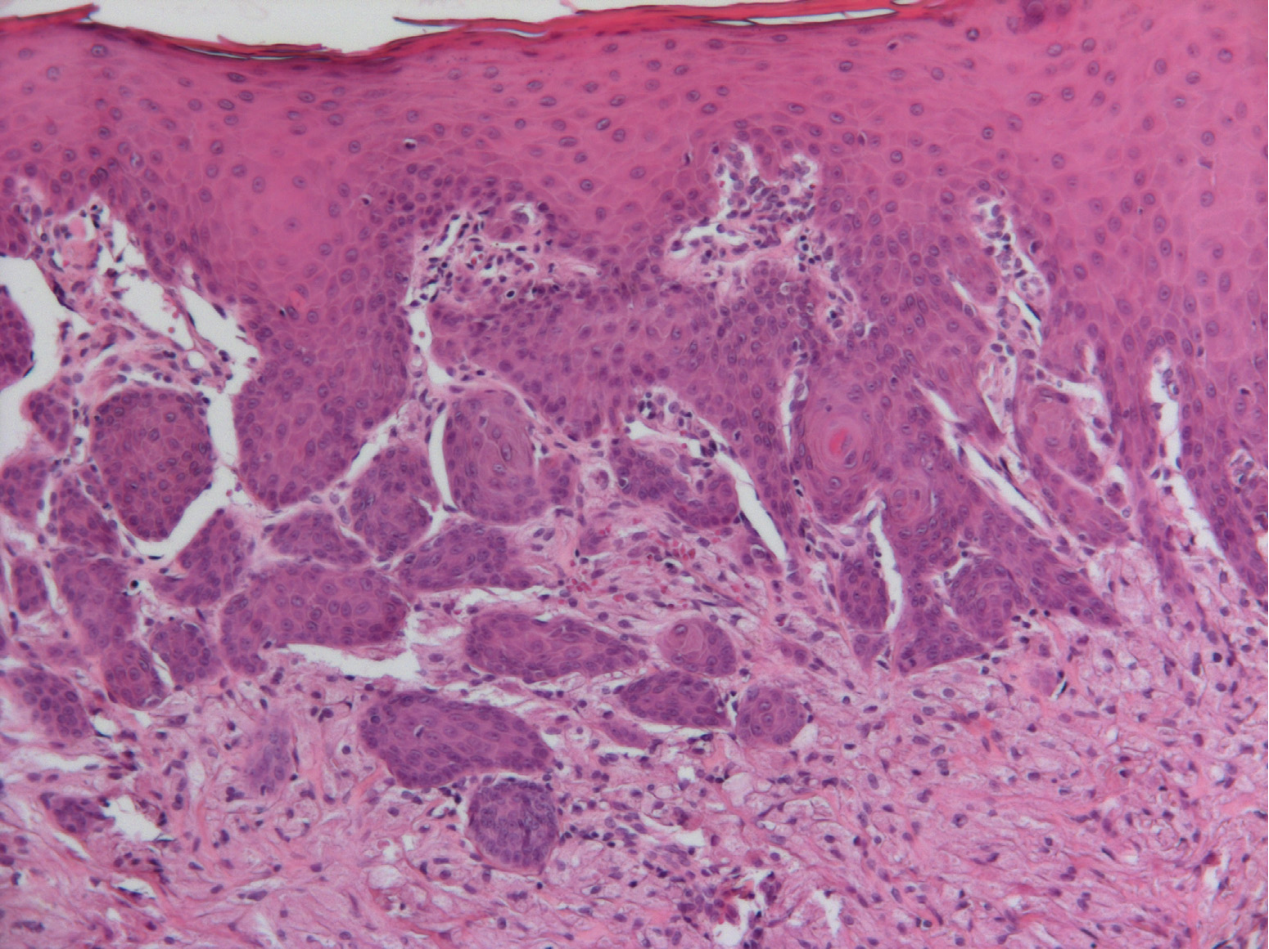
**Panoramic view of the lesion exhibiting pseudoepitheliomatous hyperplasia (hematoxylin and eosin stain, 100×)**.

A 26-year-old Caucasian man presented to the Oral Medicine out-patient clinic of the São José dos Campos Dental School, UNESP, with a 'small blister on the tongue', which he had first noted approximately three years ago. An intra-oral examination revealed a yellow nodular, sessile lesion of gummy consistency, whose texture was similar to that of the adjacent mucosa. The nodule was located on the dorsum of the tongue, measured about 1.5 cm in diameter, and was not tender to palpation (Figure 1). An excisional biopsy was performed based on the diagnostic hypothesis of GCT. Microscopic analysis showed a neoplastic lesion whose epithelium exhibited pseudoepitheliomatous hyperplasia (Figure 2). The lesion mainly consisted of large polygonal or elongated cells with clear, granular cytoplasm and an oval or round nucleus with loose chromatin, lying amidst bundles of striated muscle fibers. The diagnosis made was GCT.

### Case 2

**Figure 3:**
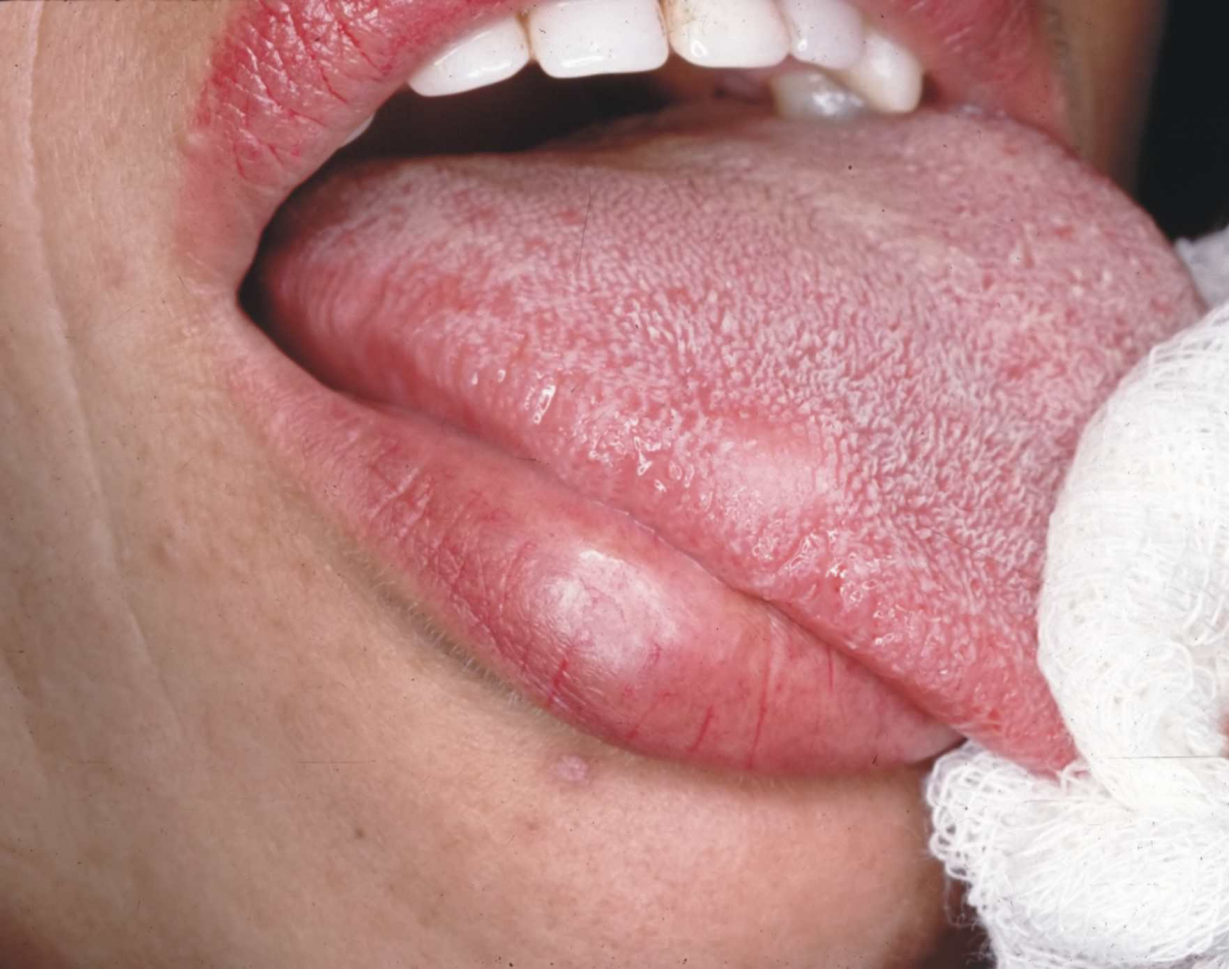
**Small nodular lesion located deep in the submucosa of the right lateral margin of the tongue**.

**Figure 4:**
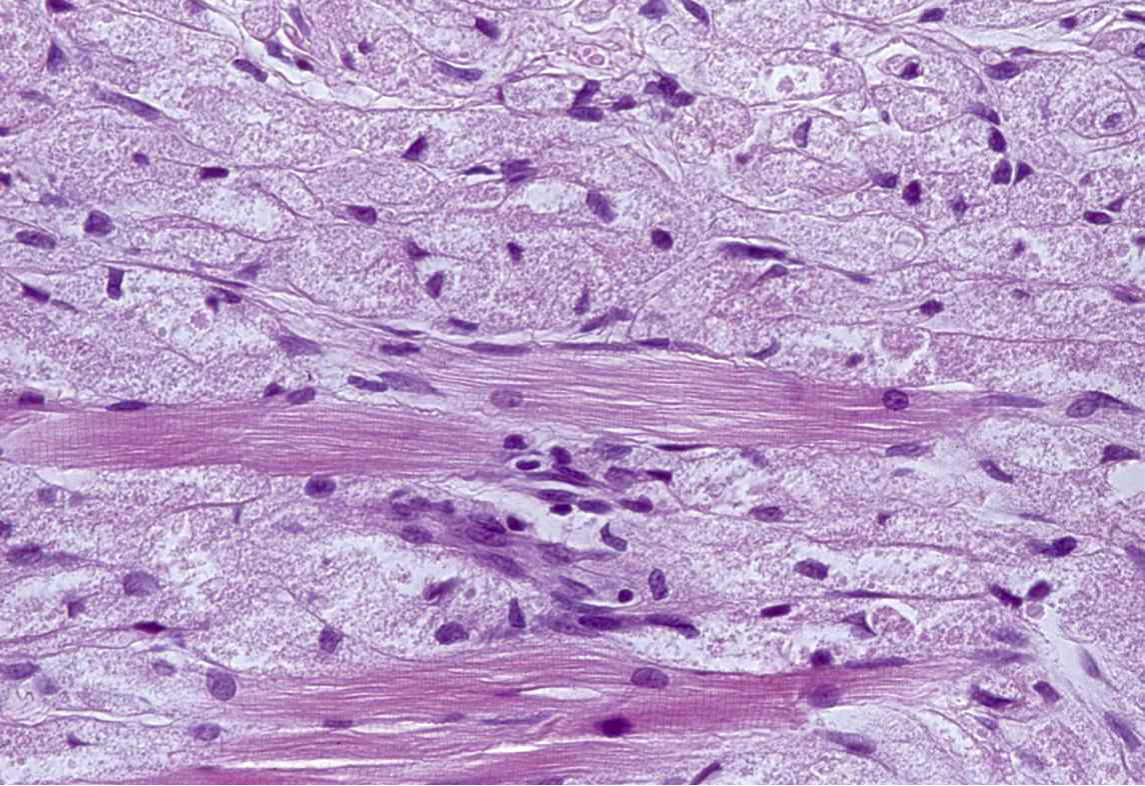
**Histopathological image of the granular cell tumor showing groups of cells with abundant granular cytoplasm**. Granular cells are present amidst bundles of striated muscle fibers (hematoxylin and eosin stain, 400×).

A 31-year-old Caucasian woman presented to the Oral Medicine out-patient clinic of the São José dos Campos Dental School, UNESP, with a five-year history of a 'painful lump on the tongue'. Intra-oral examination revealed the presence of a pale yellow nodular lesion of gummy consistency measuring approximately 0.8 cm in diameter, which was located deep in the submucosa of the right lateral margin of the tongue (Figure 3). An excisional biopsy was performed based on the differential diagnosis of neurofibroma and GCT. Large polygonal or elongated cells with clear cytoplasm and an oval or round nucleus with loose chromatin were noted in the lamina propria. Periodic acid-Schiff (PAS)-stain positive granules were detected in the cytoplasm. Striated muscle bundles and nerve fibers were observed among neoplastic cells (Figure 4). The histological diagnosis was GCT.

## Discussion

GCT is a rare tumor that can affect various regions of the body, such as the skin, soft tissues, breast, and lungs [3]. However, GCT is more frequently found in the head and neck region, which accounts for 45% to 65% of all sites affected by the tumor. Of these, 70% are located in the oral cavity, especially the tongue, oral mucosa, and hard palate [2]. Considering the wide variety of sites affected by the tumor and its variable histological presentation, a correct clinical description is fundamental. Although the etiology of GCT is still controversial, the currently most accepted hypothesis is that the tumor arises from Schwann cells or their precursors [2,4]. Immunohistochemical analysis has shown a strong and consistent positivity for protein S-100, a finding supporting the hypothesis that GCT is of peripheral nerve sheath origin [4].

In the study by Rejas *et al*. [5], the immunoprofile of GCTs showed nerve sheath differentiation, a finding lending support to a neural origin of these tumors and contributing to the establishment of a differential diagnosis between this lesion and other oral granular cell tumors, whether benign or malignant. Vered *et al*. [6] recently tested an extensive panel of antibodies to determine the true origin of this tumor. In most cases, granular cells were strongly and diffusely positive for p75, vimentin, calretinin, NKI/C3, inhibin-α, protein gene product 9.5 (PGP9.5), and protein S-100. However, the authors called attention to the fact that the antibodies stained different tissues. As a consequence, no particular cell type that would be responsible for the histogenetic origin of GCT could be identified.

GCT seems to be more prevalent among women, but a gender preference is not unanimously accepted. The tumor commonly develops between the second and sixth decade of life [7] and is rare in children [8]. Clinically, benign GCT manifests as a nodular lesion that is generally asymptomatic and solitary, although cases of multiple lesions have been reported [7,9]. The tumor presents as a pink or yellow well delimited lesion that rarely exceeds 3 cm in diameter, is covered by intact mucosa, and usually involves subcutaneous or submucosal tissues. There are reports of painful symptoms during tooth brushing, consumption of spicy foods, and bite trauma [7].

In the present report, one of our patients reported pain, whereas the tumor was asymptomatic in our other patient. Although the symptomatic lesion showed no ulceration, the deeper location of the tumor may have affected adjacent nerve fibers. In a recent study including 68 cases of GCT, Vered *et al*. [6] observed a strong association between granular cells and skeletal muscle. However, clusters of granular cells around nerve fibers were only observed in nine (21%) patients and replacement of nerves with tumor cells was noted in five of these cases.

Clinically, any nodular lesion involving oral soft tissue can be included in the differential diagnosis. Features such as consistency, color and the possible definition of lesion margins upon palpation may facilitate the establishment of diagnostic hypotheses. Histologically, GCTs are characterized by the proliferation of large polygonal neoplastic cells with cytoplasmic granules, eosinophilic cytoplasm, a small and eccentrically located nucleus, and undefined cytoplasmic limits. In some cases, the epithelium that covers the tumor exhibits pseudoepitheliomatous hyperplasia [7].

In the oral cavity, granular cells can be found in lesions other than GCT, including ameloblastoma, ameloblastic fibroma, odontogenic fibroma, odontogenic cysts, congenital epulis of the newborn, and oral lichen planus [10,11]. In view of the diversity of lesions that contain granular cells, this condition is still a matter of discussion. Marked pseudoepitheliomatous hyperplasia is observed in some cases of GCT, a fact that might lead to a false diagnosis of squamous cell carcinoma. This occurs because an incisional biopsy is performed which only involves the epithelium of the tumor surface without clear definition of granular cells. However, clinical data about the tumor and more detailed information provided by the professional who performed the biopsy may help define the diagnosis.

Although GCT is an uncommon benign neoplasm, cases of malignant GCT have been reported in the literature, including patients with more than one histological type of malignant GCT [12-14]. The coexistence of benign GCT of the tongue and squamous cell carcinoma at the same site has also been reported recently [15]. In view of this malignant potential, the tumor should be submitted to careful histopathological analysis. Data regarding tumor size, symptoms, rapid progression, invasion of adjacent structures, and the presence of regional and distant metastases are of fundamental importance for the histopathological diagnosis of benign or malignant GCT [13,15].

Surgical excision with a safety margin is the treatment of choice for GCT, although this is not always possible because the tumor lacks a capsule, a condition histologically demonstrated by an undefined cell margin. An excisional biopsy was also the treatment of choice in the present cases, but excision with tumor-free margins was not possible in case 1 because of the extent and location of the tumor. Both cases were followed-up for five years and no signs of recurrence were observed.

## Conclusions

GCT is an uncommon tumor that must be carefully and correctly diagnosed and treated.

## Consent

Written informed consent was obtained from the patients for publication of this case report and any accompanying images. A copy of the written consent is available for review by the Editor-in-Chief of the journal.

## Competing interests

The authors declare that they have no competing interests.

## Authors' contributions

ASRC and NCSC analyzed and interpreted the clinical data from our patients and wrote the manuscript. YRC performed the histopathological examination of the lesions. JDA and FB participated in the writing of the manuscript. All authors have read and approved the final version of the manuscript.

## References

[B1] Abrikossoff A Über myome, ausgehend von der quergestreiften willkürlichen Muskulatur Virch Arch 1926 260 215 233 10.1007/BF02078314

[B2] Becelli R Perugini M Gasparini G Cassoni A Fabiani F Abrikossoff's tumor J Craniofac Surg 2001 12 78 81 10.1097/00001665-200101000-00013 11314193

[B3] Speight P Barnes L, Eveson JW, Reichart P, Sidransky D Granular cell tumor World Health Organization Classification of Tumours. Pathology and Genetics. Head and Neck Tumours 2005 Lyon, France: IARC Press 185 186

[B4] Le BH Boyer PJ Lewis JE Kapadia SB Granular cell tumor: immunohistochemical assessment of inhibin-alpha, protein gene product 9.5, S100 protein, CD68, and Ki-67 proliferative index with clinical correlation Arch Pathol Lab Med 2004 128 771 775 15214825 10.5858/2004-128-771-GCTIAO

[B5] Rejas RAG Campos MS Cortes ARG Pinto DS de Sousa SCOM The neural histogenetic origin of the oral granular cell tumor: an immunohistochemical evidence Med Oral Patol Oral Cir Bucal 2011 16 e6 10 20526269 10.4317/medoral.16.e6

[B6] Vered M Carpenter WM Buchner A Granular cell tumor of the oral cavity: updated immunohistochemical profile J Oral Pathol Med 2009 38 150 159 19192059 10.1111/j.1600-0714.2008.00725.x

[B7] Collins BM Jones AC Multiple granuloma cell tumors of oral cavity: report of a case and review of the literature J Oral Maxillofac Surg 1995 53 707 711 10.1016/0278-2391(95)90178-7 7776058

[B8] Nagaraj PB Ongole R Bhujanga-Rao BR Granular cell tumor of the tongue in a 6-year-old girl - a case report Med Oral Patol Cir Bucal 2006 11 E162 164 16505796

[B9] Bomfin LE Alves FA Almeida OP Kowalski LP Perez DE Multiple granular cell tumors of the tongue and parotid gland Oral Surg Oral Med Oral Pathol Oral Radiol Endod 2009 107 e10 e13 10.1016/j.tripleo.2009.01.025 19426900

[B10] Mirchandani R Sciubba JJ Mir R Granular cell lesions of the jaw and oral cavity: a clinicopathologic, immunohistochemical and ultrastructural study J Oral Maxillofac Surg 1989 47 1248 10.1016/0278-2391(89)90718-0 2479730

[B11] Van der Meij EH van der Waal I Granular cells in oral lichen planus Oral Dis 2001 7 116 118 10.1034/j.1601-0825.2001.70209.x 11355436

[B12] Budino-Carbonero S Navarro-Vergara P Rodriguez-Ruiz JÁ Modelo-Sanchez A Torres-Garzón L Rendón-Infante JI Fortis-Sánchez E Granular cell tumors: review of the parameters determining possible malignancy Med Oral 2003 8 297 298 12937391

[B13] Chiang MJ Fang TJ Li HY Chen IH Lee FK Malignant granular cell tumor in larynx mimicking laryngeal carcinoma Am J Otolaryngol 2004 25 270 273 10.1016/j.amjoto.2004.01.005 15239036

[B14] Fanburg-Smith JC Meis-Kindblom JM Fante R Kindblom LG Malignant granular cell tumor of soft tissue: diagnostic criteria and clinicopathologic correlation Am J Surg Pathol 1998 22 779 794 10.1097/00000478-199807000-00001 9669341

[B15] Caltabiano R Cappellani A Di Vita M Lanzafame S The unique simultaneous occurrence of a squamous cell carcinoma and a granular cell tumor of the tongue at the same site: a histological and immunohistochemical study J Craniofac Surg 2008 19 1691 1694 10.1097/SCS.0b013e31818973ad 19098584

